# Knee Arthroscopy in the Era of Precision Medicine: A Comprehensive Review of Tailored Approaches and Emerging Technologies

**DOI:** 10.7759/cureus.70932

**Published:** 2024-10-06

**Authors:** Vinit Rathod, Sandeep Shrivastav, Milind R Gharpinde

**Affiliations:** 1 Department of Orthopedics, Jawaharlal Nehru Medical College, Datta Meghe Institute of Higher Education and Research, Wardha, IND

**Keywords:** biomarkers, emerging technologies, knee arthroscopy, personalized surgery, precision medicine, robotic-assisted surgery

## Abstract

Knee arthroscopy, a minimally invasive procedure, has transformed the treatment of knee pathologies by enabling direct visualization and management with minimal tissue disruption. Recent advances in precision medicine have introduced a new dimension to this field, allowing for highly individualized surgical approaches considering each patient's unique genetic, environmental, and biomechanical characteristics. This review explores the integration of precision medicine into knee arthroscopy, focusing on tailored approaches and emerging technologies. Key innovations such as robotic-assisted surgery, advanced imaging, and patient-specific instrumentation have enhanced surgical accuracy and patient outcomes, reduced recovery times, and minimized postoperative complications. The review also examines the role of biomarkers in guiding personalized treatment strategies, including ligament reconstructions, meniscal repairs, and cartilage restoration, which are now being refined to cater to the specific needs of individual patients. While the benefits of these innovations are clear, there are challenges to widespread adoption, including cost, resource allocation, and the need for further research to validate the efficacy of precision-driven approaches in knee arthroscopy. Moreover, the ethical considerations surrounding personalized medicine, such as patient privacy and genetic data usage, must also be addressed. Despite these barriers, the future of knee arthroscopy in the era of precision medicine holds great promise, with ongoing developments in artificial intelligence, genomics, and biomarker discovery poised to further refine patient-centered care. This comprehensive review provides valuable insights into how precision medicine reshapes knee arthroscopy, offering a glimpse into the future of more targeted and effective orthopedic interventions. By embracing these advancements, surgeons and healthcare providers can ensure optimal outcomes for patients undergoing knee arthroscopy.

## Introduction and background

Knee arthroscopy has emerged as a cornerstone of minimally invasive surgical techniques in orthopedics. This procedure allows for direct visualization, diagnosis, and management of a wide range of knee joint pathologies using small incisions, reducing the need for more invasive surgical approaches [1]. Traditional open knee surgeries, though effective, often result in extended recovery times, higher risks of complications, and increased postoperative pain. In contrast, knee arthroscopy significantly minimizes these drawbacks, offering patients faster recovery, less postoperative discomfort, and improved cosmetic outcomes. With its application in treating conditions like meniscal tears, anterior cruciate ligament (ACL) reconstructions, and cartilage repair, knee arthroscopy has gained widespread acceptance for its efficiency and positive patient outcomes across various demographics [2].

In recent years, the concept of precision medicine has revolutionized many healthcare fields, and orthopedic surgery is no exception. Precision medicine, often described as personalized or individualized medicine, aims to tailor medical treatments and interventions based on a patient's unique genetic makeup, environmental exposures, and lifestyle factors. This approach marks a significant shift from the traditional one-size-fits-all model of care, promoting more targeted and effective treatments [3]. Precision medicine is highly relevant in the context of knee arthroscopy as it opens up new avenues for customizing surgical interventions. For example, surgeons can now consider specific patient factors such as genetic predisposition to certain conditions, molecular profiles of the knee joint, and even biomechanical characteristics when planning surgical procedures. This has led to the development of more refined, patient-specific techniques, reducing the risk of complications and enhancing recovery outcomes [4].

This comprehensive review aims to explore the intersection of precision medicine and knee arthroscopy, with a particular focus on the tailored approaches and emerging technologies that are transforming the field. By examining the historical evolution of knee arthroscopy, the principles underpinning precision medicine, and the innovative technologies that have recently emerged, this review aims to highlight how these advancements are reshaping patient care. Additionally, it will address the current challenges and limitations of integrating precision-driven strategies in knee arthroscopy and discuss future research and clinical application directions. In doing so, the review seeks to provide valuable insights into how personalized, technology-enhanced surgical interventions can further improve patient outcomes in orthopedic practice.

## Review

Evolution of knee arthroscopy

Knee arthroscopy has significantly transformed from basic techniques to advanced, precision-driven approaches. This evolution can be categorized into three primary areas: historical development, advancements in surgical techniques and equipment, and the shift from conventional to precision-driven methodologies [5]. The origins of arthroscopy date back to the 1800s, with the development of the cystoscope for bladder examination, which laid the groundwork for visualizing internal structures. In 1912, Severin Nordentoft became the first to apply an endoscope to the knee joint, marking a pivotal moment in joint visualization [6]. The 1920s saw Kenji Takagi develop the first arthroscope specifically designed for knee examination, although it was not initially suitable for widespread clinical use. His work primarily focused on diagnosing tuberculous knees. The practical application of arthroscopy expanded significantly in the 1950s when Masaki Watanabe introduced fiber optics, revolutionizing the field. Watanabe performed the first arthroscopic meniscectomy in 1962, further solidifying arthroscopy's role in orthopedic surgery. By the 1970s, modern arthroscopic systems began incorporating cold light sources, enhancing visibility during procedures, and extending arthroscopic techniques to other joints beyond the knee [6]. Technological innovations have been instrumental in advancing knee arthroscopy. Today’s systems utilize ultrahigh-definition imaging, providing superior visualization of joint structures and significantly improving diagnostic and surgical precision. The emergence of robotic-assisted surgery allows for enhanced accuracy through three-dimensional surgical planning and real-time adjustments during procedures. Moreover, minimally invasive techniques have become standard practice, employing smaller incisions that result in reduced tissue damage, less postoperative pain, and quicker recovery times than traditional open surgeries [7].

Instrumentation has also seen remarkable advancements. New implant technologies with sensors deliver real-time data on joint function and recovery progress, enabling more personalized postoperative care. Additionally, advances in three-dimensional printing allow for the creation of patient-specific implants that closely match individual anatomical structures, improving surgical outcomes [8]. The transition toward precision medicine in knee arthroscopy emphasizes individualized treatment plans based on a patient's unique anatomy and specific conditions. This approach leverages data from advanced imaging and monitoring technologies to effectively tailor interventions. Enhanced recovery protocols have evolved alongside these advancements; with minimally invasive techniques and precise surgical methods, recovery regimens now focus on accelerated rehabilitation. Patients benefit from shorter recovery times and improved functional outcomes due to targeted physical therapy supported by real-time data from smart devices [9]. Looking ahead, the future of knee arthroscopy is set for further advancements with emerging technologies such as nanotechnology and regenerative medicine. These innovations aim to enhance joint repair processes while minimizing the need for invasive procedures, potentially transforming how orthopedic surgeons approach knee pathologies [8].

Principles of precision medicine in knee arthroscopy

Precision medicine represents an innovative approach to healthcare, tailoring medical treatments to the individual characteristics of each patient. This paradigm shift moves away from a one-size-fits-all model, strongly emphasizing individualization, data-driven insights, and a holistic perspective. Individualization entails customizing treatments based on a patient's genetic makeup, lifestyle, and environmental factors. Data-driven insights leverage advanced technologies, such as genomics and proteomics, to gather information that informs treatment decisions. Furthermore, a holistic approach considers multiple biological, psychological, and social influences to optimize patient outcomes [10]. In knee arthroscopy, personalized treatment plans are increasingly utilized to enhance surgical outcomes. This process begins with a thorough preoperative assessment that employs advanced imaging techniques and a comprehensive patient history to develop tailored surgical strategies. Surgeons can implement customized surgical techniques, utilizing minimally invasive approaches and robotic-assisted systems that adapt to the patient's unique anatomical features [11]. Such an approach ensures precise interventions while also minimizing recovery times. Postoperative monitoring is equally crucial; smart implants and wearable technology track recovery metrics in real time, allowing for adjustments in rehabilitation protocols based on individual progress [11]. Integrating molecular biology into knee arthroscopy transforms treatment paradigms by applying genomics, proteomics, and biomarker-driven interventions. Genomics aids in understanding genetic predispositions that can guide decisions regarding surgical interventions and postoperative care. For instance, patients with specific genetic markers may respond differently to certain treatments, enabling more informed choices about surgery or conservative management [12]. Proteomics studies proteins involved in joint health and helps identify biomarkers to predict surgical outcomes or complications. This information facilitates tailored interventions that mitigate risks associated with knee surgeries. Moreover, identifying biomarkers related to inflammation or cartilage degradation can lead to targeted therapies that enhance recovery and reduce the necessity for invasive procedures [12]. Principles of precision medicine in knee arthroscopy are illustrated in Table 1.

**Table 1:** Principles of precision medicine in knee arthroscopy ACL: anterior cruciate ligament; AR: augmented reality; VR: virtual reality; PSI: patient-specific instrumentation

Principle	Description	Applications in knee arthroscopy
Genomic and proteomic profiling [13]	Utilizes patient-specific molecular data, such as genetic and protein markers, to predict disease risk, treatment responses, and healing potential	Identifying patients with genetic predispositions for delayed healing or complications, customizing pharmacologic interventions to improve outcomes
Personalized biomechanical analysis [14]	Involves the use of advanced imaging and motion analysis to assess individual joint kinematics and load distribution for tailored surgical approaches	Enhances surgical precision in ligament reconstruction and cartilage repair by providing detailed insight into the patient’s unique biomechanics, optimizing graft placement and surgical technique
Robotics and navigation systems [15]	Leverages robotic assistance and navigation systems for real-time intraoperative guidance, enhancing accuracy and minimizing human error	Used in ACL reconstruction, meniscal repair, and complex cartilage procedures, offering enhanced precision in graft placement, cutting, and soft tissue management
AR and VR [16]	Provides immersive visualization tools for preoperative planning and intraoperative assistance, allowing surgeons to visualize patient-specific anatomy in 3D	Improves the accuracy of procedures by overlaying patient-specific anatomical details during arthroscopy, assisting in meniscal repairs, chondroplasty, and cartilage restoration
Bioprinting and tissue engineering [17]	Involves creating custom biological grafts and implants tailored to the patient’s anatomy and pathology using 3D bioprinting and tissue engineering techniques	Enables personalized meniscal and cartilage restoration by producing grafts closely mimicking native tissue, improving long-term durability and function
Smart implants and wearable sensors [18]	Real-time data collection through implants and sensors that monitor healing progress, joint movement, and load distribution during recovery	Provides continuous postoperative monitoring, allowing for early detection of complications and the ability to adjust rehabilitation protocols based on patient-specific recovery data
PSI [19]	Custom instruments are designed based on preoperative imaging to match the patient's anatomy for increased surgical precision	Tailored ACL reconstruction and meniscal repair with reduced surgical time, improved graft positioning, and minimized risk of postoperative complications
Biological augmentation [20]	Use growth factors, stem cells, and biological agents to enhance tissue healing and regeneration	Enhances cartilage repair and meniscal healing by delivering biological agents directly to the injury site, promoting faster and more effective recovery

Patient-specific approaches in knee arthroscopy

The evolution of knee arthroscopy underscores the importance of tailoring surgical techniques and rehabilitation programs to individual patient profiles. This personalized approach enhances outcomes and minimizes complications, making it a crucial aspect of modern orthopedic care [21]. Patient profiling is fundamental in determining the most effective treatment strategies. Age plays a significant role, as younger patients may present with different knee conditions and have varying recovery expectations compared to older individuals, who often have more comorbidities that can complicate surgical outcomes. Additionally, a patient's activity level is critical; active individuals may require more aggressive interventions to restore functionality, whereas sedentary patients might benefit from less invasive options that prioritize safety and recovery [22]. Comorbidities also significantly influence surgical decisions. Conditions such as obesity, diabetes, and cardiovascular diseases can complicate both the surgery itself and the recovery process. By assessing these factors, surgeons can customize their approaches to ensure that the selected techniques align with the patient’s overall health status, enhancing safety and effectiveness [23]. Recent advancements in biomarker research enable surgeons to tailor surgical techniques based on individual biological markers. This customization can optimize surgical outcomes by identifying inflammation levels that indicate the extent of tissue damage, guiding the choice of surgical interventions. Additionally, understanding a patient’s biological response to surgery can inform decisions regarding regenerative techniques or specific rehabilitation protocols [24]. Moreover, technologies such as 3D printing facilitate the creation of patient-specific implants that better fit individual anatomies. This personalized approach improves integration with natural tissues and enhances overall function after surgery, leading to improved long-term outcomes [25]. Rehabilitation following knee arthroscopy is critical for recovery and long-term success. Individualized rehabilitation programs can include tailored exercise regimens designed by physical therapists based on preoperative assessments. These regimens cater to each patient's activity level and personal goals, ensuring a more effective recovery process [26]. The integration of smart technology also plays a vital role in rehabilitation. Wearable devices can monitor recovery metrics such as range of motion and activity levels, allowing for real-time adjustments to rehabilitation plans. Furthermore, telehealth integration enhances patient engagement by providing remote monitoring and consultations, making it easier for patients to adhere to rehabilitation protocols [27]. Patient-specific approaches in knee arthroscopy are illustrated in Table 2.

**Table 2:** Patient-specific approaches in knee arthroscopy PSI: patient-specific instrumentation; ACL: anterior cruciate ligament; AI: artificial intelligence; MRI: magnetic resonance imaging; CT: computed tomography; AR: augmented reality; VR: virtual reality; ACI: autologous chondrocyte implantation; PRP: platelet-rich plasma

Approach	Description	Key benefits	Example technologies/techniques
Genomic and proteomic profiling [28]	Analyzing genetic and protein data to identify individual risk factors for complications and healing responses	Tailored pharmacological interventions, optimized rehabilitation, and reduced postoperative complications	Genetic testing and proteomic markers
Personalized biomechanical analysis [29]	Utilizing patient-specific joint kinematics and load distribution to customize surgical techniques	Improved accuracy in surgical planning, better alignment, and reduced risk of mechanical failure	3D motion analysis and computational modeling
PSI [30]	Customized surgical tools based on patient’s anatomical data from preoperative imaging	Enhanced precision in procedures such as ACL reconstruction, reduced surgical time, and improved implant positioning	MRI/CT-based custom instruments
Robotics and navigation systems [31]	Robotic-assisted surgery with real-time feedback and navigation for precise execution of procedures	Increased accuracy, reduced human error, and enhanced precision in ligament reconstruction, graft placement, and cartilage repair	Robotic-assisted surgery platforms and intraoperative navigation systems
AR and VR [32]	Real-time visualization of anatomical structures during surgery using AR and preoperative planning through VR	Enhanced precision in complex procedures, improved surgeon training, and better preoperative planning for customized interventions	AR glasses and VR surgical planning platforms
Bioprinting and tissue engineering [33]	Creating patient-specific grafts and implants that replicate native cartilage or meniscus	Personalized cartilage restoration and meniscal repair with longer lasting results	3D bioprinting and tissue-engineered scaffolds
Smart implants and wearable sensors [34]	Real-time monitoring of joint movement, load, and healing progress through wearable or implantable devices	Data-driven postoperative rehabilitation, early detection of complications, and personalized rehabilitation plans	Smart knee implants and wearable motion sensors
Cartilage restoration with precision medicine [35]	Customizing cartilage repair techniques based on patient-specific molecular and biomechanical data	Enhanced durability and functionality of repaired cartilage and better selection of candidates for specific treatments	ACI, osteochondral allograft transplantation, growth factors, or stem cell therapies
Meniscal repair with biological augmentation [36]	Tailoring meniscal repair techniques using patient-specific healing potential, often augmented with biological agents	Improved meniscal preservation, faster healing, and reduced risk of future meniscal degeneration	All-inside repair systems, growth factors, and PRP injections

Emerging technologies enhancing knee arthroscopy

Knee arthroscopy is undergoing a significant transformation, driven by innovative technologies that enhance precision, accuracy, and overall patient outcomes. Among these advancements, robotic-assisted systems stand out for their ability to improve surgical precision. These robotic platforms enable surgeons to perform procedures more accurately, making real-time adjustments based on the patient's unique anatomy. Additionally, integrating artificial intelligence (AI) into surgical planning is revolutionizing the field. AI can analyze extensive datasets to assist surgeons in making informed decisions, thereby optimizing treatment plans tailored to individual needs [11]. Another groundbreaking area is using 3D imaging and virtual reality (VR) tools. Advanced imaging technologies provide high-definition, three-dimensional views of the knee joint, facilitating better presurgical planning and enhancing the surgeon's understanding of the joint's complex anatomy. Furthermore, VR tools are being incorporated into training programs for surgeons, allowing them to practice procedures in a simulated environment. This immersive experience enhances surgical skills and prepares surgeons for complex cases without the risks associated with real-life operations [37]. Intraoperative navigation systems are also making a significant impact on knee arthroscopy. These systems offer real-time feedback during surgery, providing enhanced surgical field visualization. This technology ensures accurate placement of instruments and implants, thereby reducing the likelihood of complications and improving overall surgical outcomes [38]. Finally, nanotechnology and wearable devices are emerging as essential components in postoperative care. Wearable sensors can monitor patients' recovery progress after surgery by tracking activity levels and joint function. These data are transmitted to healthcare providers for ongoing assessment, allowing for timely interventions if necessary. Additionally, advancements in nanotechnology may enhance healing processes by delivering targeted therapies directly to affected tissues, potentially speeding up recovery times and improving outcomes [39]. Integrating these cutting-edge technologies into knee arthroscopy represents a significant leap forward in orthopedic surgery. Enhancing precision and personalizing care promises to improve patient experiences and outcomes in knee surgeries [8]. Emerging technologies enhancing knee arthroscopy are summarized in Table 3.

**Table 3:** Emerging technologies that enhance knee arthroscopy ACL: anterior cruciate ligament; AR: augmented reality; VR: virtual reality; AI: artificial intelligence; MRI: magnetic resonance imaging; CT: computed tomography

Technology	Description	Applications in knee arthroscopy	Key benefits
Robotic-assisted surgery [40]	Robotic systems provide real-time feedback and assist in precision surgery, such as ligament reconstruction	ACL reconstruction, meniscal repair, and complex cartilage restoration	Increased accuracy, reduced human error, optimized graft placement, and enhanced precision
AR [41]	AR overlays real-time digital information onto the surgical field, enhancing the surgeon's view of the anatomy	Meniscal repair, cartilage restoration, and ACL reconstruction	Improved intraoperative visualization, enhanced precision in soft tissue procedures, and better navigation during surgery
VR [42]	VR is used for surgical training, preoperative planning, and simulation of knee arthroscopy	Preoperative planning, surgical simulations, and training of knee arthroscopy procedures	Enhanced surgical training, better preoperative visualization, and improved surgeon preparedness
3D bioprinting [43]	3D bioprinting allows for the creation of patient-specific grafts and tissue scaffolds for cartilage or meniscus repair	Cartilage restoration and meniscal repair	Customizable tissue regeneration, improved integration of grafts, and long-term durability of repairs
Smart implants [34]	Smart implants provide real-time data on knee joint performance, load distribution, and healing progress after surgery	ACL reconstruction, knee replacement monitoring, and postarthroscopic healing tracking	Real-time data collection, personalized rehabilitation, and early detection of complications
Wearable sensors [44]	Sensors worn on the body or integrated into rehabilitation devices to monitor joint movement and recovery	Postoperative rehabilitation and real-time tracking of knee function after surgery	Data-driven recovery protocols, personalized rehab plans, and better monitoring of patient progress
3D motion analysis [45]	This technology uses high-resolution cameras and sensors to analyze knee joint kinematics during movement	Preoperative assessment of joint function, biomechanics analysis, and postinjury functional assessment	Precise understanding of joint dynamics and customized surgical planning based on individual biomechanics
AI algorithms [46]	AI algorithms analyze large datasets to optimize surgical planning and postoperative rehabilitation strategies	Preoperative risk stratification, optimization of postoperative rehab programs, and prediction of surgical outcomes	Enhanced decision-making, personalized treatment plans, and data-driven postoperative care
High-resolution imaging technologies [47]	Advanced imaging, such as 3D MRI or CT, for detailed visualization of knee joint structures	Preoperative planning, intraoperative guidance in cartilage repair, meniscal surgeries, and ligament reconstruction	Improved diagnostic accuracy, better visualization of soft tissues and cartilage, and early detection of pathologies
Nanotechnology in implants [48]	Nanoscale materials and coatings for knee implants to improve biocompatibility and durability	Joint resurfacing, cartilage restoration, and meniscus replacement	Enhanced integration with natural tissue, reduced inflammation, and longer lasting implants

Tailored surgical techniques

Knee arthroscopy is experiencing a transformative evolution, characterized by tailored surgical techniques that enhance the precision and effectiveness of treatments. Innovations in ligament repair and reconstruction, particularly with techniques such as bridge-enhancing ACL repair (BEAR) and internal bracing (IB), are leading this evolution. The BEAR technique facilitates the natural healing of the ACL by using an implant that bridges the torn ends of the ligament, combined with the patient’s blood, to promote healing. This approach not only preserves the original attachments of the ACL but also has the potential to lead to faster recovery times and improved patient satisfaction compared to traditional reconstruction methods. Similarly, IB augments ACL reconstruction by utilizing suture tape as a secondary stabilizer, enhancing graft integration and enabling earlier rehabilitation without increasing the risk of graft failure [49]. In meniscal repair, personalized treatment pathways are becoming increasingly prevalent. Surgeons are leveraging advanced 3D imaging technologies to create detailed models of a patient's knee, allowing customized surgical approaches tailored to individual anatomical variations and specific injury patterns. This precision in surgical planning can lead to better outcomes and reduced complications, ensuring that each patient receives care that closely aligns with their unique needs [43]. Cartilage restoration is also benefiting from innovative techniques that emphasize customization. Regenerative medicine approaches, such as stem cell therapy and tissue engineering, aim to harness the body’s natural healing processes to restore cartilage. These methods have the potential to reduce the need for invasive surgeries or artificial implants. Furthermore, 3D printing technology creates patient-specific implants that fit individual anatomical requirements perfectly. This level of customization not only improves implant integration but also minimizes complications commonly associated with standard implants [20]. The shift toward patient-specific instrumentation is further enhancing surgical precision in knee arthroscopy. Surgeons can now utilize advanced imaging techniques to develop highly accurate 3D models of a patient's joint, which inform personalized treatment plans and implant designs. This tailored approach significantly enhances surgical accuracy and improves postoperative outcomes [50]. Tailored surgical techniques in knee arthroscopy are summarized in Table 4.

**Table 4:** Tailored surgical techniques in knee arthroscopy PSI: patient-specific instrumentation; ACL: anterior cruciate ligament; PRP: platelet-rich plasma; ACI: autologous chondrocyte implantation; PCL: posterior cruciate ligament; HTO: high tibial osteotomy

Surgical technique	Description	Application in knee arthroscopy	Key benefits
PSI [19]	Customized surgical tools designed from preoperative imaging, tailored to the patient’s specific anatomy	ACL reconstruction, meniscal repair, and osteochondral transplantation	Enhanced precision, reduced surgical time, and improved implant positioning
All-inside meniscal repair [51]	A minimally invasive technique that uses smaller incisions and fewer sutures, tailored to the type and location of the tear	Meniscal tear repair	Minimally invasive, reduced recovery time, and preservation of meniscal tissue
Biological augmentation of cartilage repair [52]	Use of growth factors, stem cells, or PRP to enhance healing in cartilage restoration tailored to patient pathology	Cartilage restoration techniques such as microfracture and ACI	Accelerated healing, improved cartilage durability, and enhanced tissue regeneration
ACI [53]	Personalized approach using the patient's cartilage cells for repair	Treatment of focal cartilage defects	Long-term durability, patient-specific tissue regeneration, and better cartilage integration
Osteochondral autograft transplantation [54]	Transfer of cartilage from a non-weight-bearing area to the damaged area, tailored to patient-specific cartilage defects	Focal cartilage defect repair	Personalized cartilage restoration, reduced risk of rejection, and faster integration
Ligament reconstruction with custom grafts [55]	Use of patient-specific grafts (e.g., hamstring or patellar tendon) for ligament repair, with personalized graft placement	ACL and PCL reconstructions	Enhanced graft durability, better anatomical fit, and optimized biomechanics after surgery
Partial knee replacement [56]	Customized to treat specific compartments of the knee affected by arthritis or injury, sparing healthy tissues	Patients with localized knee osteoarthritis or injury	Preservation of healthy tissue, reduced recovery time, and more natural joint movement
HTO [57]	Tailored realignment surgery that shifts weight-bearing load away from the damaged part of the knee	Patients with knee malalignment or early-stage arthritis	Delays need for full knee replacement, preserves joint function, and reduces pain
Custom 3D-printed implants [58]	Personalized implants made using 3D printing technology to fit individual patient anatomy	Joint resurfacing, and partial or full knee replacements	Perfect anatomical fit, reduced implant failure, and enhanced joint function
Minimally invasive ACL reconstruction [59]	ACL reconstruction using smaller incisions and patient-specific grafts	ACL injury treatment	Quicker recovery, less postoperative pain, reduced scarring, and tailored graft placement for better functional outcomes

Challenges and limitations in precision knee arthroscopy

The integration of precision medicine into knee arthroscopy encounters numerous challenges and limitations that impede its widespread adoption. Key concerns include barriers to personalized approaches, costs, resource allocation, and ethical considerations related to biomarker-driven surgery [60]. One of the foremost obstacles to adopting personalized methods in knee arthroscopy is the lack of standardization. Without established protocols for implementing these innovative techniques, variability in practice can result in inconsistent outcomes. This inconsistency makes it difficult for healthcare providers to justify deviating from traditional approaches [61]. Additionally, surgeons may require specialized training to effectively utilize new technologies, which can discourage institutions from adopting these advancements, particularly in resource-limited settings. Moreover, resistance to change within the medical community poses a challenge, as established surgical practices are often deeply ingrained. Many practitioners may hesitate to embrace new methodologies if they perceive them as unproven or overly complex [61]. Cost is another significant barrier associated with precision knee arthroscopy. Advanced technologies like robotic systems and high-definition imaging often entail substantial upfront investments. Healthcare facilities must carefully assess whether the potential benefits of these technologies justify their costs, which can be a significant hurdle for many institutions. Furthermore, ongoing maintenance and upgrades of sophisticated equipment can strain budgets, compelling hospitals to balance the need for cutting-edge technology against financial constraints [62]. Economic viability is also a concern; studies have indicated that the cost-effectiveness of arthroscopic procedures is sometimes questionable compared to nonoperative treatments. In certain instances, including arthroscopy does not significantly enhance outcomes relative to its costs, raising further doubts about its economic justification in standard practice [62]. Ethical and clinical issues surrounding biomarker-driven surgery add another layer of complexity. The reliance on biomarkers to guide surgical decisions raises questions about their predictive validity. If these biomarkers do not accurately reflect patient outcomes, there is a risk of inappropriate surgical interventions that could potentially harm patients.

Additionally, personalized approaches dependent on advanced diagnostics may exacerbate disparities in healthcare access [63]. Patients in underresourced settings may lack access to necessary tests or technologies, leading to unequal treatment opportunities. Informed consent also becomes more intricate with biomarker-driven approaches; the complexity requires clear communication with patients regarding potential risks and benefits. Ensuring that patients fully understand these factors can be challenging, especially when discussing novel technologies that are still evolving [63]. The challenges and limitations in precision knee arthroscopy are summarized in Table 5.

**Table 5:** Challenges and limitations in precision knee arthroscopy AI: artificial intelligence

Challenge/limitation	Description	Impact on knee arthroscopy	Potential solutions
High cost of advanced technologies [64]	Precision tools and technologies, such as robotics, 3D printing, and smart implants, are expensive	Limits accessibility for patients and increases the overall cost of treatment	Development of cost-effective technologies and insurance coverage expansion
Limited access to genomic and proteomic data [65]	Not all patients have access to genomic testing, limiting personalized treatment planning	Hinders the ability to tailor surgical approaches based on molecular data	Integration of affordable genetic screening in routine clinical settings
Lack of clinical validation [66]	Many emerging technologies lack large-scale clinical trials to validate their long-term efficacy	Slows the adoption of novel precision-based techniques in routine clinical practice	Conducting more comprehensive, multicenter clinical trials
Steep learning curve for surgeons [67]	Technologies like robotics and AI require specialized training and experience	Limits widespread use of precision technologies, especially in low-resource settings	Increased access to training programs, hands-on workshops, and virtual simulations for surgeons
Interoperability issues between systems [68]	Lack of standardization among different technologies (e.g., robotic systems, imaging, and navigation platforms)	This can result in compatibility problems during surgery, reducing efficiency and precision	Development of standardized protocols and better integration between systems
Increased surgery time [69]	The use of advanced technologies may increase the time taken for surgery due to the need for setup and precision tasks	Prolonged operating times can increase risks like infection and longer recovery periods for patients	Refining procedures to streamline technology use and decrease time
Patient selection criteria [11]	Not all patients are ideal candidates for precision-based approaches (e.g., genomic therapies or robotic surgeries)	Some patients may not benefit from precision techniques, leading to inconsistent outcomes	Development of clearer guidelines for patient selection
Data privacy and ethical concerns [70]	Collecting and storing large amounts of genetic and biomechanical data raises privacy concerns	This may deter patients from opting for precision medicine-based approaches due to fear of data misuse	Stronger data protection regulations, informed consent processes, and transparent data use policies
Technological failure or malfunction [71]	Reliance on technology increases the risk of failure or malfunction during surgery	This can lead to complications if robotic systems or navigation tools fail mid-procedure	Establishing reliable backup systems and improved quality control measures
Variation in postoperative outcomes [72]	Despite precision techniques, variability in patient healing and response to surgery still exists	This can lead to inconsistent results in terms of recovery time, pain relief, and long-term success	Continued research into individual factors that affect postoperative recovery
Patient awareness and expectations [73]	Patients may have limited knowledge of precision arthroscopy, leading to unrealistic expectations	This can result in dissatisfaction if outcomes do not meet patient expectations	Better patient education and setting realistic expectations during preoperative consultations

Future directions and research

Integrating AI, machine learning, and big data into knee arthroscopy presents substantial potential for advancing orthopedic surgery. These technologies can analyze extensive datasets derived from patient histories, imaging results, and surgical outcomes to uncover patterns that enhance both preoperative planning and postoperative care [74]. For example, AI algorithms can assist surgeons in predicting potential complications, optimizing surgical techniques, and personalizing rehabilitation protocols based on individual patient profiles. By leveraging big data analytics, healthcare providers can make more informed decisions, drawing insights from a wider array of patient experiences and outcomes [74]. Another promising area of research is the application of genetic testing in preoperative planning for knee arthroscopy. Understanding genetic predispositions related to drug metabolism and surgical recovery can lead to more tailored anesthetic and postoperative medication strategies [75]. Pharmacogenomics, which examines inherited differences in drug metabolism among individuals, can inform medication choices, potentially minimizing adverse effects and improving recovery times. Integrating genetic testing results into electronic health records (EHRs) allows healthcare providers to receive real-time alerts regarding genetic risks associated with specific medications, thereby enhancing perioperative outcomes [75]. Long-term studies focusing on patient outcomes following personalized knee arthroscopy are crucial for validating the effectiveness of these tailored approaches. Research should aim to assess how individualized treatment plans, considering factors such as genetic profiles, lifestyle choices, and specific knee conditions, impact recovery times, pain management, and overall patient satisfaction [76]. Such studies will provide critical data to support the evolution of precision medicine in orthopedics, ultimately leading to improved surgical techniques and enhanced patient care [76]. Future directions and research in precision knee arthroscopy are summarized in Table 6.

**Table 6:** Future directions and research in precision knee arthroscopy AI: artificial intelligence; CRISPR: clustered regularly interspaced short palindromic repeats; MRI: magnetic resonance imaging

Future direction/research area	Description	Potential impact on knee arthroscopy	Current challenges
AI integration [77]	Advanced AI algorithms for preoperative planning, surgical decision-making, and postoperative care	Improved accuracy in diagnosis, personalized surgical plans, and data-driven rehabilitation strategies	Need for large-scale data integration and ethical concerns regarding decision-making
Machine learning for predictive outcomes [78]	Using machine learning models to predict patient outcomes based on preoperative data and surgery type	Better prediction of recovery times, risk of complications, and likelihood of successful outcomes	Requires access to extensive patient data and validation of predictive models
Development of biocompatible smart implants [79]	Creating implants that monitor joint health, healing, and response to stress in real time	Continuous monitoring of postoperative healing, improved rehabilitation, and early detection of complications	High cost and technical complexity in developing durable, biocompatible smart implants
3D-printed personalized implants [80]	Research into fully customized implants created through 3D printing tailored to the patient's specific anatomy	Increased precision in joint replacement and cartilage repair, and reduced implant failure rates	High costs, regulatory challenges, and lack of long-term data on the effectiveness of custom implants
Gene therapy for cartilage repair [81]	Exploring gene-editing techniques like CRISPR to promote cartilage regeneration and repair	Potential to prevent or reverse cartilage damage, reducing the need for joint replacement	Ethical concerns, limited clinical trials, and unknown long-term effects
Tissue engineering and regenerative medicine [82]	Research into tissue scaffolds, stem cells, and bioprinting to regrow damaged cartilage or meniscus tissue	Long-term cartilage and meniscal restoration, potentially eliminating the need for artificial implants	Complex manufacturing processes, high cost, and variable patient response
Wearable health monitoring devices [83]	Research into wearable devices that continuously monitor joint motion, pressure, and rehabilitation progress after surgery	Real-time data collection to adjust rehabilitation protocols and detect complications early	Data privacy concerns, integration with clinical systems, and patient adherence to wearing devices
Robotics with haptic feedback [84]	Development of robotic systems that provide surgeons with tactile feedback during arthroscopy	Enhanced precision in surgeries, better handling of delicate tissues, and reduced error rates	High cost, steep learning curve for surgeons, and complex integration into surgical workflows
Enhanced imaging techniques [85]	Research into new imaging modalities such as high-resolution MRI and intraoperative ultrasound	Better visualization of cartilage, ligaments, and soft tissues for more accurate diagnosis and real-time surgical guidance	High cost and limited access to advanced imaging technologies
Personalized rehabilitation programs [86]	Development of data-driven, individualized rehabilitation programs based on patient’s surgical data and biomechanical feedback	Improved recovery times, reduced risk of reinjury, and personalized care based on real-time monitoring	Requires integration of wearable technology and AI for real-time data interpretation
Nanotechnology in cartilage repair [87]	Research into using nanoscale materials to repair cartilage or prevent degeneration	Potential for long-lasting cartilage repair, reduced inflammation, and improved healing outcomes	Limited clinical research, high development costs, and the need for regulatory approval
Telemedicine and remote surgery assistance [88]	Expanding telemedicine for postoperative care and exploring robotic surgery assistance remotely	Improved patient access to specialized care, enhanced monitoring of recovery, and potential for expert guidance during surgery	Security concerns with remote technology, availability of internet infrastructure, and surgeon adaptability

## Conclusions

The integration of precision medicine into knee arthroscopy represents a significant advancement in orthopedic surgery, paving the way for more personalized and effective interventions. By leveraging the principles of precision medicine, surgeons can now tailor their approach to individual patient profiles, considering genetic, molecular, and biomechanical factors. Emerging technologies, such as robotic-assisted surgery, advanced imaging, and patient-specific instruments, further enhance the precision and outcomes of knee arthroscopic procedures. These innovations improve the accuracy of diagnosis and treatment, minimize complications, and accelerate recovery. However, despite the promising benefits, challenges such as cost, accessibility, and the need for further clinical validation remain. As research continues to advance, the future of knee arthroscopy in the era of precision medicine holds the potential to offer even more refined, patient-centered care. This comprehensive review underscores the importance of continued exploration and adoption of these tailored approaches, ensuring patients receive the most advanced and effective treatments possible.
